# Mindfulness, Compassion, and Stigma Toward People with Mental Illness: A Cross-Sectional Study of Medical and Nursing Students in the Czech Republic

**DOI:** 10.1192/j.eurpsy.2026.10624

**Published:** 2026-07-31

**Authors:** M. Janoušková, E. Zakreski, L. Bankovská Motlová

**Affiliations:** 1 Division of Medical Psychology, Third Medical Faculty, Charles University; 2 Department of Psychology and Life Sciences, Faculty of Humanities, Charles University, Prague; 3 Center for Sexual Health and Interventions, National Institute of Mental Health, Klecany, Czech Republic

## Abstract

**Introduction:**

Mindfulness can be defined as intentional, non-judgmental awareness of the present moment, including inner states and external environment. It is both a trainable skill and a personality trait, associated with stress reduction, enhanced well-being, and emotion regulation. Compassion refers to the emotional response of caring for another who is suffering, combined with a motivation to help. While mindfulness and compassion have been linked to reduced prejudice and intergroup bias, their role in addressing stigma toward people with mental illness remains understudied. This is particularly relevant in healthcare, where stigma can negatively affect treatment, recovery, and quality of life.

**Objectives:**

The aim of this study was to examine the associations between mindfulness, self-compassion, compassion, and stigma toward people with mental illness among medical and nursing students.

**Methods:**

This cross-sectional study was conducted among students of a medical faculty (n=240). Data were collected online. Validated Czech versions of the Self-Compassion Scale (SCS), Santa Clara Brief Compassion Scale (SCBCS), Five Facet Mindfulness Questionnaire (FFMQ), Perceived Stress Scale (PSS), and the Opening Minds Scale for Health Care Providers (OMS-HC) were administered. Multiple linear regression analyses were performed with OMS-HC total scores as the dependent variable, and FFMQ, SCS, SCBCS, and PSS as predictors, adjusting for gender, grade, field of study, mindfulness practice, and experience with mental illness.

**Results:**

The average OMS-HC total score was 31.5, with subscale means of 13.3 for attitudes, 7.8 for disclosure, and 10.4 for social distance, suggesting overall low levels of stigma. Regression analyses showed that experiencing mental illness (B=–0.202, p=.002) and greater compassion (B=–0.224, p=.001) were significantly associated with lower stigma. In contrast, higher mindfulness scores were unexpectedly associated with greater stigma (B=0.202, p=.002). Self-compassion and perceived stress were not significant predictors. The overall model explained 15.6% of the variance in stigma.

**Conclusions:**

This study highlights the destigmatizing potential of compassion and lived experience with mental health problems. Surprisingly, mindfulness was associated with higher stigma, which raises questions about the differential role of specific mindfulness facets. These findings suggest that not all forms of mindfulness function uniformly, and that general attentional awareness (trait mindfulness) may differ in its effects from compassion-oriented mindfulness practices. These findings underscore the relevance of integrating compassion-based approaches into medical education to promote more inclusive attitudes toward people with mental illness.

**Disclosure of Interest:**

None Declared

